# Supplementary material for: Diagnostic Value of Conventional PET Parameters and Radiomic Features Extracted from 18F-FDG-PET/CT for Histologic Subtype Classification and Characterization of Lung Neuroendocrine Neoplasms
Source: Biomedicines. 2021 Mar 10;9(3):281. doi: 10.3390/biomedicines9030281 (PMC8001140; doi:10.3390/biomedicines9030281)
Supplement: Supplementary file 1 [file biomedicines-09-00281-s001.pdf]

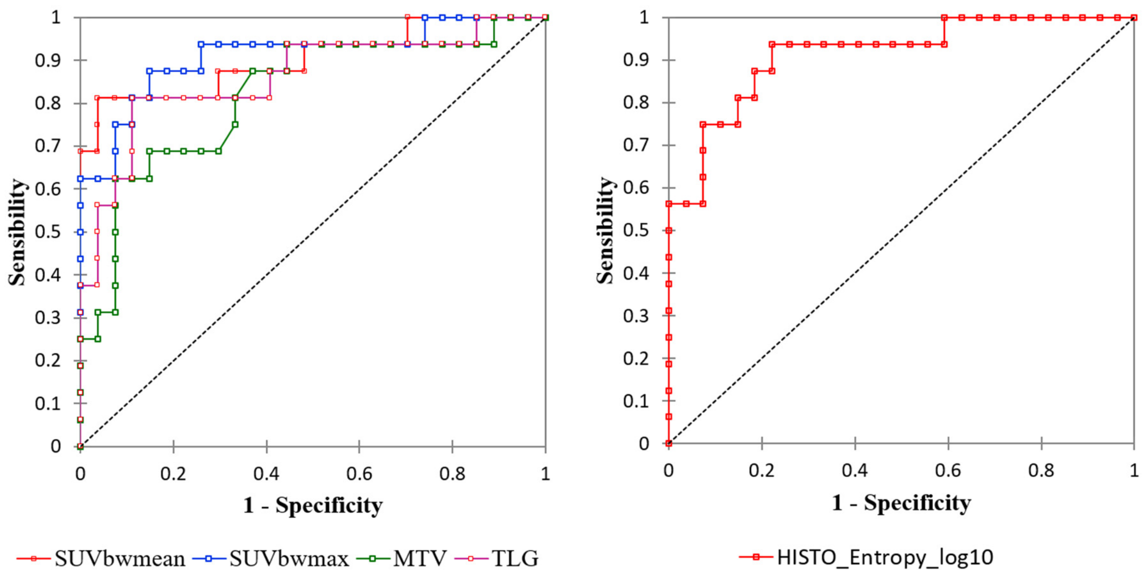

Supplementary Figure S1. ROC curve analysis of the conventional and volumetric PET parameters (left) and Histo_Entropy_log10 (right) excluding the 3 patients with small-cell lung cancer (SCLC).

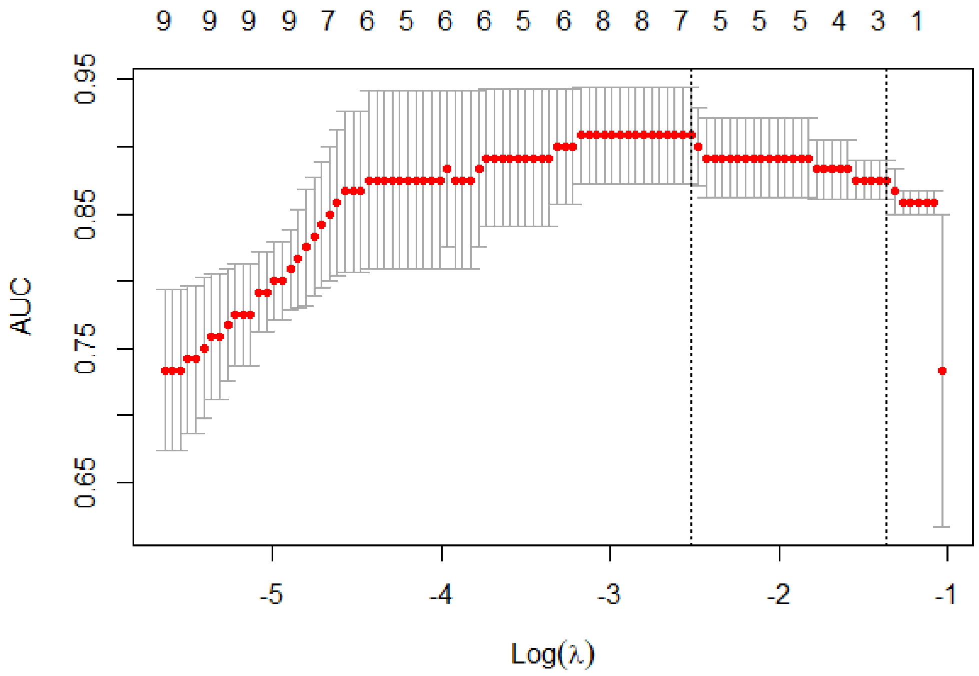

Supplementary Figure S2. Radiomics features selection using LASSO in the population excluding the 3 patients with SCLC.
